# Science With Purpose: 50 Years of the Institute of Occupational Medicine

**DOI:** 10.3389/fpubh.2022.924678

**Published:** 2022-06-23

**Authors:** Anthony Seaton, John W. Cherrie, Hilary Cowie, Robert J. Aitken

**Affiliations:** ^1^Institute of Occupational Medicine, Edinburgh, United Kingdom; ^2^University of Aberdeen, King's College London, London, United Kingdom

**Keywords:** multidisciplinary research, occupational health, airborne particles, respiratory protection, ergonomics

## Abstract

The Institute of Occupational Medicine (IOM) was founded in 1969 by the then UK National Coal Board to complete its nation-wide epidemiological study of lung disease in coal miners, the Pneumoconiosis Field Research. The results quantified risks in the industry and were influential across the world in setting preventive standards. The research, based on epidemiology, was multidisciplinary from the start, and the IOM's broad scientific expertise was applied across many other industries with an increasing focus on environmental measurement and ergonomics. In 1990, as the coal industry declined, IOM became a self-funding research charity with a strong commercial arm. It has expanded its research, often with European collaborators and funding from governments, and has achieved wide recognition. This has most recently been applied during the pandemic in areas of hospital ventilation, personal protection, and viral exposure research, illustrating IOM's ability to respond to new environmental or occupational challenges.

## Origins

The nationalization of the UK coal industry in 1946 revealed a huge epidemic of chronic lung disease in miners. With funding from the UK Government, the industry began an epidemiological research programme in selected collieries on 50,000 miners, recording symptoms, lung function, radiology, study of selected lungs *post mortem*, and detailed estimates of exposure to respirable coal dust. The research was intended to address two questions, (i) how much and what kinds of dust caused pneumoconiosis and (ii) what action needed to be taken to prevent disablement of miners? To coordinate and analyse the data and oversee the research, in 1969 the National Coal Board founded the Institute of Occupational Medicine (IOM) in Edinburgh as a research charity. A detailed account of its history was published in 2019 and this provides further detail (1).

From the early years, IOM's staff comprised specialists in occupational medicine, pathology, statistics, physics, chemistry, and ergonomics. Its research was and remains multi-disciplinary. The early results were the first worldwide to show quantitative relationships between dust exposure and risk of pneumoconiosis in coal miners (2, 3). These led to formulation of science-based preventive occupational standards in the UK, USA and elsewhere.

## The First 20 Years: 1969–89

The unique contribution of the early research was in quantification of risks in epidemiology. This led not only to understanding of pneumoconiosis but also to quantifying the increased risk of chronic obstructive lung disease and emphysema (COPD) in coal miners (4, 5), related conditions commonly caused by tobacco smoking. This contributed to recognition of COPD for industrial disability compensation in the UK. The research established the IOM's international reputation in occupational medicine and hygiene.

Alongside the coal research, IOM developed a programme of research into asbestos diseases, the other outstanding occupational risk issue of these decades. The emphasis was on understanding experimentally what made asbestos fibers so uniquely dangerous, causing asbestosis, lung cancer and pleural mesothelioma. The answer related to the diameter and length of the fibers and their solubility in lung fluid once inhaled (6, 7). This research enabled more reliable measurement of asbestos in relation to risk, both for epidemiology and in workplace hygiene control. Furthermore, it led to ways of assessing the potential hazard of new non-asbestos mineral fibers being introduced into industry and to a research programme into such fibers (8).

The experience and reputation in epidemiology gained by IOM led to contracts from many different industries including steel in relation to cancer risks, brickworks, and quarries and opencast coal in relation to lung risks. Major international projects included research into risks of shale mining for oil production in the USA (9), assessment of occupational exposures in the mineral wool industries across Europe (10), and studies of risks related to a volcanic eruption in Montserrat (11). In the UK, IOM also carried out unique studies of lung problems in wool workers (12), agricultural use of pesticides (13), dust exposure in the London Underground (14), and exposures in relation to PVC production (15). The coal studies also led to important contributions to understanding the risks of exposure to silica and to the setting of a standard to reduce risks of silicosis (16).

Beside the early research into occupational epidemiology and pathology were two important programmes, occupational hygiene, and ergonomics. The hygiene research addressed the need better to quantify toxic exposures, especially in relation to dust, and how to protect against them. New methods of measuring dust and airborne asbestos fibers developed by IOM exposure scientists have been adopted as standard approaches throughout the world, most notably with the IOM inhalable dust sampler (17), and the Walton-Beckett microscope eyepiece graticule for measuring asbestos and other fibers (18).

Measurement of workplace hazards led naturally to studies of improved methods of protection, especially personal protective equipment (PPE), including programmes on respirators (19), masks and building ventilation that were later to find substantial application in the COVID-19 pandemic.

The IOM was a significant contributor to ergonomics, at a time when this was a relatively young discipline, developing a leading role internationally. The UK Ergonomics Research Society (the oldest such society worldwide, and now the Chartered Institute of Ergonomics and Human Factors) is only 10 years older than ergonomics at the IOM itself. This research had started in the complex activities required in coal mining but spread to developing means of improving the physical and psychological conditions associated with more mundane activities such as keyboard work, bending and lifting, and stress (20–24). Occupational hygiene and ergonomics were both to become fundamental to the IOM's continued existence in more recent years.

By the late 1980s the UK coal industry was in terminal decline and doubt was cast on the IOM's future. However, IOM scientists, many with high international reputations, were publishing over 50 refereed papers annually, and contributing to multiple Government and European committees, and the Institute was counted among the best-known in the world. Sufficient non-Coal Board grant funding was being obtained for some research to continue and a service function based primarily on occupational hygiene and ergonomics together with teaching courses had been developed that made survival appear possible. Costs were cut by reducing staff, all the most senior scientists and the Director taking redundancy and being succeeded by their deputies. Additional short-term funding from the Coal Board to ensure completion of European contracts, and grants from the Colt Foundation and the mineral fiber industries were obtained and in 1989 IOM became a self-funding research charity, supported by an enlarged commercial section to sell its services.

## The Independent Institute 1990–2020

The move to financial independence was a bold one, as few if any research institutes exist without core funding from government or other benefactors. The solution to this was to expand the commercial side of the organization while maximizing grant income for the charitable research. Many of the research areas lent themselves to provision also of consultancy and measurement services, notably with respect to ergonomics and occupational risk and safety. The 1990s were a period when there was increasing recognition of musculoskeletal and psychological problems in workplaces, and these provided opportunities for the IOM. At the same time, new opportunities came from collaborative research in Europe in ergonomics, the application of exposure science in epidemiology, and in the new nanotechnologies.

### Ergonomics and Occupational Hygiene

IOM continued its important research in both musculoskeletal disorders and psychological stress, the major causes of work-related absence (20, 25, 26). Other studies embraced protection from heat and noise, safe design of machines and workplaces, and safe handling of materials (21, 23, 24, 27). Starting from the earlier work in mines, IOM developed a research interest in personal protection, and has made significant contributions to the protection of firefighters, attracting grants from the Health and Safety Executive and the Home Office, and contributing to US action in the aftermath of the terrorist attacks known as 9/11. The IOM has won three awards from the Chartered Institute of Ergonomics for its research over the years. In addition, IOM scientists have much experience in evaluating the effectiveness of respirators and face masks (28–30).

Research continued on the issue of estimating exposures to toxic substances. As a fundamental part of the European research project in the synthetic fiber manufacturing industry, new mathematical modeling approaches in estimating historic workplace exposures were developed; this has subsequently been applied to produce tools to estimate human exposure for European chemical regulation (10). Pioneering work on the measurement of skin exposure and inadvertent ingestion exposure in workplaces has led to IOM becoming one of the main international centers of expertise in these aspects of exposure science (31, 32).

### Air Pollution and Nanoparticles

IOM scientists had from 1990 been involved in Government advisory committees on air pollution, stemming from their work in particle inhalation. From this interest arose the concept that the cardiac effects of pollution were caused by nanoparticles derived from burning fossil fuel (33). This coincided with a rising interest in the industrial and scientific use of nanomaterials. A report to Government by the Royal Society and Royal Academy of Engineering drew attention to the need for research in possible hazards in nanotechnologies and European grants subsequently became available for this (34).

With its background in particle toxicology and risk assessment, IOM was well placed to address some of these challenges, particularly those relating to the protection of individuals who might be exposed to these new materials. Its work, with many international collaborators, had defined the physical properties that make particles toxic, including asbestos fibers, which are often of a nanosized diameter. Studies of dust regarded as non-toxic had shown that they might overload defense mechanisms in the lung, leading to an important conceptual advance in toxicology based on the surface area of dust particles rather than the mass (35, 36). These became of critical importance in understanding the toxic properties of nanoparticles. Building on this background, IOM embarked on a strategy to develop nanotechnology risk as both a scientific programme and a business opportunity, seeking to understand and explain risks and how they could be reduced. The proactive approach taken demonstrated that it was possible to raise the profile of a whole area of science, build capacity and credibility, and move the international agenda (37). It enabled IOM to develop in innovative ways, from new ideas about science communication to thinking in a new paradigm where risk research was key to supporting technological innovation.

The expertise of IOM scientists was applied during the COVID-19 pandemic in assisting the NHS in testing the effectiveness of room ventilation in hospital wards and providing advice about the use of respiratory protection. Research was carried out to measure the virus in the air and on surfaces in healthcare settings and developed a mathematical model to predict the effectiveness of different control strategies in reducing the infection risk for staff. IOM is part of the UK PROTECT COVID-19 National Core Study on transmission and environment, assisting with transmission modeling and investigations of risk in specific sectors, such as the food processing industry.

## Fifty Years of Multidisciplinary Science

The past fifty years have provided many challenges, not least to an organization doing medical scientific research initially supported by a dying industry. So far IOM has survived two major coal strikes, two major economic crashes, the loss of its core financial support, Brexit, and now at least four waves of the greatest pandemic since 1918. IOM's history provides many valuable lessons to inform its future success. Of overriding importance has been a commitment to practical and applied science with the purpose of improving the health of workers and the wider population. This ethos, combined with the commitment of its staff, many of whom can look back with pride over decades of work with us, has been the foundation of our success.

Understanding and managing the risks from inhaled particles has been a central theme but the need to adapt both the topics addressed and the methods used has been critical. Evolution of the organizational structure with emphasis on varying types and models of funded work at different times has been necessary to ensure IOM's continued existence (1). Underpinning this has been its enduring commitment to its values of independence, impartiality, integrity, quality and sustainability.

## The Changing World of Work

IOM's primary concern is in the prevention of occupational and environmental causes of ill health. Since the promotion and maintenance of good health are among the most fundamental and important global challenges, there are many opportunities to apply its scientific and advisory knowledge. During its 50 years, the world of work has changed dramatically. In the West in particular, there is a decline in employment in manufacturing and extractive industries, and an increase in what is thought of as office work. Increasingly people work at home.

The emergence of a 24/7 culture has disrupted traditional patterns of work-life balance and social support mechanisms. The use of artificial intelligence, big data, robotics and the internet is growing rapidly. Millions of people spend their entire working day in front of a computer or other interface device. There are fewer large companies, lots of small to medium enterprises, and many millions of self-employed individuals. People change their jobs and careers often in their working life. There is a huge rise in the “gig” economy, in which people have temporary jobs or are doing separate paid pieces of work, rather than working on a regular contract, and there is much more part-time working.

As western economies age, retirement age also rises, leading to a workforce with increased vulnerabilities, often managing chronic disease or reduced mobilities. Hazards in the modern workplace can also relate to the way that work is organized rather than specific agents, and the consequential harm may be more psychological than physical. Construction is growing rapidly, particularly in developing economies, often with new techniques and materials. In industrial, scientific, and high-technology manufacturing, people may be exposed to new hazards. In developing economies, millions are still employed in extraction, construction, and manufacturing, often in conditions which have become rare in the West. Exposure to dust, asbestos and agricultural chemicals remains a major issue worldwide.

## The Changing Environment

There is an irony in that the IOM arose from the humanitarian desire of a state-owned coal industry to protect the health its workforce, while the product of that workforce, coal for combustion, has subsequently proved to be the basis of the world's greatest threat to civilisation's survival, climate change, and one of its major causes of ill health, air pollution (38). Along with warfare, social injustice, and pestilence these may be seen by many as the fourth metaphorical horseman of the apocalypse.

IOM was early to recognize the commonality of occupational and environmental disease, requiring the same scientific approaches to their investigation and amelioration. In many developing countries infectious diseases remain the most important causes of early death, but the current COVID-19 pandemic caught Europe unprepared. The IOM's expertise in PPE and hospital ventilation (for preventing cross-infection) played an important part in advising governments and in saving lives. However, infection aside, in most countries, adult morbidity and mortality is usually dominated by non-communicable conditions, including heart disease, cancer, diabetes and chronic musculo-skeletal and respiratory diseases. Environmental air pollution, indoor and out, is now recognized as a significant contributor world-wide to death in people with these chronic diseases, including dementia (39).

The United Nations Sustainable Development Goals (SDGs) call on countries to mobilize efforts to end poverty, fight inequalities and tackle climate change, whilst ensuring that no country is left behind. They recognize that ending poverty must go hand-in-hand with strategies that build economic growth and address a range of social needs including education, health, social protection, and job opportunities, whilst tackling climate change and protecting a diverse environment.

## Vision for the Future

The world is changing rapidly, perhaps more so now than at any time in IOM's history. In response, IOM continues to adapt and evolve to stay relevant, add value and realize its purpose. IOM's vision is to remain a globally recognized organization that engages and empowers its staff to deliver its mission of contributing to a healthy and sustainable world. Its focus is the application of science to key risk issues of importance, “*to maximize the relevance, benefits and impact of our work”*. This includes development of its existing areas of occupational and workplace health, exposure science, nanotechnology, citizen science, human factors, hospital ventilation, and wellbeing. Within these areas, new topics are emerging such as shiftwork and cancer, dementia in professional sports people, and robotics and automation, where IOM is already beginning to work and achieve prominence. The international dimension of IOM's work is also important, since the issues it addresses are worldwide.

IOM's origin addressed the challenge of answering two questions (i) how much and what kinds of dust caused pneumoconiosis and (ii) what action needed to be taken to prevent disablement of miners. Addressing these questions has opened many opportunities in the workplace and general environment for IOM to build on what has already been achieved. These questions when adapted to new industrial and environmental scenarios and the associated health risks remain pertinent. Future success will depend on IOM's ability to innovate and remain relevant to the changing needs of society.

## Author's Note

All research contracts throughout IOM's history have included a clause ensuring freedom to publish results in the scientific literature.

## Data Availability Statement

The original contributions presented in the study are included in the article/supplementary material, further inquiries can be directed to the corresponding author.

## Author Contributions

AS wrote the first draft of the manuscript. RA, JC, and HC contributed to manuscript revision. All authors read and approved the submitted version. All authors contributed to the content of the manuscript which is an overview of the scientific work of the IOM, in which they all had substantial involvement.

## Conflict of Interest

The authors declare that the research was conducted in the absence of any commercial or financial relationships that could be construed as a potential conflict of interest.

## Publisher's Note

All claims expressed in this article are solely those of the authors and do not necessarily represent those of their affiliated organizations, or those of the publisher, the editors and the reviewers. Any product that may be evaluated in this article, or claim that may be made by its manufacturer, is not guaranteed or endorsed by the publisher.
